# Genetic Interactions Affect Lung Function in Patients with Systemic Sclerosis

**DOI:** 10.1534/g3.119.400775

**Published:** 2019-11-06

**Authors:** Anna Tyler, J. Matthew Mahoney, Gregory W. Carter

**Affiliations:** * The Jackson Laboratory, 600 Main St. Bar Harbor, ME,; † Department of Neurological Sciences, Larner College of Medicine, University of Vermont, Burlington, VT, and; ‡ Department of Computer Science, University of Vermont, Burlington, VT

**Keywords:** Epistasis, Systemic Sclerosis, Scleroderma, Wnt Signaling

## Abstract

Scleroderma, or systemic sclerosis (SSc), is an autoimmune disease characterized by progressive fibrosis of the skin and internal organs. The most common cause of death in people with SSc is lung disease, but the pathogenesis of lung disease in SSc is insufficiently understood to devise specific treatment strategies. Developing targeted treatments requires not only the identification of molecular processes involved in SSc-associated lung disease, but also understanding of how these processes interact to drive pathology. One potentially powerful approach is to identify alleles that interact genetically to influence lung outcomes in patients with SSc. Analysis of interactions, rather than individual allele effects, has the potential to delineate molecular interactions that are important in SSc-related lung pathology. However, detecting genetic interactions, or epistasis, in human cohorts is challenging. Large numbers of variants with low minor allele frequencies, paired with heterogeneous disease presentation, reduce power to detect epistasis. Here we present an analysis that increases power to detect epistasis in human genome-wide association studies (GWAS). We tested for genetic interactions influencing lung function and autoantibody status in a cohort of 416 SSc patients. Using Matrix Epistasis to filter SNPs followed by the Combined Analysis of Pleiotropy and Epistasis (CAPE), we identified a network of interacting alleles influencing lung function in patients with SSc. In particular, we identified a three-gene network comprising *WNT5A*, *RBMS3*, and *MSI2*, which in combination influenced multiple pulmonary pathology measures. The associations of these genes with lung outcomes in SSc are novel and high-confidence. Furthermore, gene coexpression analysis suggested that the interactions we identified are tissue-specific, thus differentiating SSc-related pathogenic processes in lung from those in skin.

Systemic sclerosis (SSc), or scleroderma, is a complex autoimmune disease associated with significant morbidity and mortality. It is characterized by widespread fibrosis of skin and internal organs, as well as vasculopathy (Denton and Khanna 2017). The most common cause of death in SSc is lung disease (Steen and Medsger 2007). Roughly a quarter of people with SSc develop interstitial lung disease, which involves lung fibrosis, vascular hyperreactivity, and inflammation (Solomon *et al.* 2013). Another 7–13% of patients develop pulmonary arterial hypertension, which is characterized by vascular injury and occlusion, vasoconstriction, and dysregulated angiogenesis (Solomon *et al.* 2013). Both conditions lead to reduced lung function and increased risk of death. The pathogenesis of lung disease in SSc is not understood well enough for development of specific treatments, and current treatments rely primarily on non-specific immune suppression (Cappelli *et al.* 2015). There is a need to identify new molecular drivers of lung disease in SSc, as well as how these drivers interact with other genes to influence pathogenesis.

A standard approach to discovering molecular drivers of lung disease in SSc is to identify genetic variants associated with lung outcomes. Genetic studies have been tremendously successful in identifying genetic variants associated with SSc and its complications. In a reflection of the complexity of the disease, variants in over 200 genes have been implicated in SSc risk and progression (Yu *et al.* 2010), which has greatly increased our understanding of the development of SSc (Mayes 2012; Agarwal 2010; Agarwal and Reveille 2010) and may aid in personalized disease monitoring and treatment (Assassi *et al.* 2013). The next step in this line of inquiry is to incorporate genetic complexity into models that determine how variants interact with each other to influence disease. By explicitly modeling genetic interactions, or epistasis, we can build understanding of how molecular pathways work in concert to drive SSc pathology.

Initial studies of genetic interactions in SSc have been promising. Epistasis between polymorphisms in the HLA region and cytokines has been shown to predict SSc risk (Beretta *et al.* 2008a), development of severe ventilatory restriction (Beretta *et al.* 2008b), and digital ulcer formation (Beretta *et al.* 2010) in SSc patients. However, progress in this search is limited by a number of challenges. The rarity of the disease and its clinical heterogeneity add to difficulties present in all human genetic studies, such as low minor allele frequencies and the large number of potentially relevant variants. Non-parametric tests such as Multifactor Dimensionality Reduction (MDR) (Hahn *et al.* 2003) have been successful in identifying the interactions that have been identified thus far (Beretta *et al.* 2008a,b 2010). These findings suggest additional, complementary interaction analyses may further dissect the genetic complexity of SSc and other common diseases.

Here we present a novel approach that increases power to detect genetic interactions in human genome-wide association studies (GWAS). We previously developed the Combined Analysis of Pleiotropy and Epistasis (CAPE) to model epistatic interactions in model organisms (Tyler *et al.* 2013; Carter *et al.* 2012). CAPE increases power to detect and interpret genetic interactions by combining information across multiple traits into a single consistent model. We have demonstrated its ability to identify novel genetic interactions not detectable by other methods (Tyler *et al.* 2014, 2016). For this study, we combined CAPE with a filtering step, which filtered the SNPs to those most likely to be involved in genetic interactions. We used Matrix Epistasis (Zhu and Fang 2018), an ultra-fast method for exhaustively testing epistasis in genome-wide SNP data. Candidate SNP pairs were then analyzed with CAPE and significance was assessed with permutation tests.

We applied this approach to genetic and clinical data from a cohort of patients with SSc (dbGaP accession phs000357.v2.p1). To capture aspects of lung disease and autoimmunity, we analyzed two measures of lung function, forced vital capacity (FVC) and diffusion lung capacity (DLC), as well as two autoantibody staining patterns: centromeric, and nucleolar. Anti-centromere autoantibodies (ACA) are associated with pulmonary hypertension, and anti-nucleolar autoantibodies (ANA) are associated with progressive interstitial lung disease and pulmonary arterial hypertension (Betteridge *et al.* 2016). Thus, there may be common genetic pathways underlying both autoantibody status and lung function that we can identify by analyzing all four traits simultaneously.

## Materials and Methods

### Methods workflow

All code used for computational analyses in this paper are available in a supplemental workflow available at https://github.com/annaLtyler/SSc_Epistasis_Workflow.

### Genetic and clinical data

We obtained genotype and phenotype from the database of Genotypes and Phenotypes (dbGaP) Accession phs000357.v1.p1.

The full data comprised 833 SSc patients, including 741 females and 85 males. Genotypes were measured at 601,273 SNPs using the Human610_Quadv1_B platform. Patients were assessed for the presence of multiple autoantibodies and lung function tests were performed. Values for autoantibody phenotypes were 1 (present) and 0 (absent), and values for test of lung function included forced vital capacity and diffusion lung capacity. Both measurements were represented as the percent of the predicted value based on factors such as age and sex (Agarwal *et al.* 2009).

We analyzed two lung function traits, percent predicted forced vital capacity (FVCP) and percent predicted diffusion lung capacity (DLCP) as well as two autoantibody patterns, anti-centromere (ACA) and anti-nucleolar (ANA). There were 416 patients (369 females and 47 males) with measurements for all four traits, and all subsequent analyses used only these patients.

### Expression data

We obtained gene expression data from the Gene Expression Omnibus (GEO) (Edgar *et al.* 2002; Barrett *et al.* 2013). The data set we used (accession number GSE76808) compared gene expression in lung biopsies taken from patients with SSc and interstitial lung disease (ILD) to biopsies of unaffected tissue from lung cancer patients undergoing surgical resection (Christmann *et al.* 2014). This is the only data set available that compares SSc lung to normal lung. The available data were $log_{2}$ normalized, thus we did not perform further normalization. Group-wise comparisons were performed with two-tailed Student’s two-sample *t*-tests.

### SNP filtering

We first reduced to 243,662 SNPs with minor allele frequency (MAF) ≥0.1, a relatively high cutoff due to our pair-wise testing strategy. Because analysis of all pairwise combinations of the filtered SNPs was computationally infeasible, we further filtered the SNPs to 1500 SNPs that were likely to participate in genetic interactions influencing the four traits. The number 1500 was selected such that the CAPE pipeline would take about 24 hr to run. For this filter we used Matrix Epistasis (Zhu and Fang 2018), an ultra-fast method of calculating interactions between SNPs. Using Matrix Epistasis, we were able to calculate interaction scores for all SNP pairs exhaustively for each of the four traits. We used a permissive *p* value significance threshold of $p<1\times 10^{6}$ to limit the false discovery inflation cause by trait-based SNP selection. We then used an iterative scheme to identify SNPs that were epistatic and pleiotropic (affecting more than one trait) based on this significance threshold. In the first stage of the search, we identified SNP pairs that interacted significantly ($p<1\times 10^{-6}$) to influence more than one trait. There were 52 pairs of SNPs that interacted to affect both lung traits. No other SNP pairs influenced more than one trait. In the next stage of the search, we looked for individual SNPs that were in epistatic interactions affecting more than one trait, regardless of the interacting partner. For example, SNP1 might interact with SNP2 to influence ANA, but interact with SNP3 to influence ACA. SNP1 affects both traits through interactions, but by interacting with different partners. In this case, we keep SNP1, but discard SNPs 2 and 3. We searched in this way for SNPs that influenced all four traits as part of interactions, and then all three traits as part of interactions, and so on until we had selected 1500 SNPs that were potentially epistatic and pleiotropic. For this filtering step as well as for CAPE, we used a dominant coding for the SNPs; *i.e.*, loci homozygous for the major allele were coded as 0 and loci that were either heterozygous or homozygous for the minor allele were coded as 1. This coding allows for maximum representation of each two-locus genotype combination, which increases power to detect dominant effects, but reduces power to detect recessive or additive effects.

### Combined Analysis of Pleiotropy and Epistasis (CAPE)

We identified genetic interactions between SNPs using Combined Analysis of Pleiotropy and Epistasis (CAPE) (Tyler *et al.* 2013; Carter *et al.* 2012). This method combines information across multiple traits to infer directed genetic interactions. Combining information across traits not only increases power to detect genetic interactions, but also allows inference of the allele-to-allele direction of the interaction, which can be either positive (enhancing) or negative (suppressing). We have applied this analysis to multiple model organisms including yeast (Carter *et al.* 2012), fly cell lines (Carter 2013), and mice (Philip *et al.* 2014; Tyler *et al.* 2014, 2017, 2016). This is the first application to a human cohort.

As a preliminary analysis, we tested whether SNPs previously associated with SSc could be recapitulated in this small cohort. We performed linear regression to associate each SNP with each of the four traits.

We then decomposed the autoantibody and lung traits to four orthogonal eigentraits (ETs) using singular value decomposition (SVD) (Figure 2). This step concentrates trait variance into composite ETs potentially improving the ability to map weak genetic effects that are otherwise distributed across traits. We analyzed the first two ETs, which explained 71% of the variance across the four phenotypes (Figure 2A). These ETs contrasted each of the autoantibodies with the other autoantibody and the two lung traits in turn (Figure 2A).

We performed linear regression on SNP pairs and each ET. To avoid testing SNPs in linkage disequilibrium, we did not test any SNP pairs whose Pearson correlation coefficient exceeded an arbitrary, stringent cutoff of 0.5. Out of 1,125,750 possible pairs between the 1500 SNPs plus the covariate sex, 1,125,593 SNP pairs (>99.9%) passed this criterion. We also assessed population structure in the patient cohort. Population structure, caused by heterogeneous relatedness among individuals in a cohort, can artificially inflate the association between SNPs and traits. To test whether population structure was influencing our association tests, we compared the quantile normalized distribution of the $-log_{10}\left(p\right)$ from association tests with each trait to the distribution of quantile normalized *p* values under the null hypothesis (Fig S1). There was no systematic inflation of the estimates of significance. We therefore did not correct for population structure in this study.

After performing all pairwise regressions, we recast the regression coefficients to CAPE parameters describing allele-to-allele influences. This process is described in detail elsewhere (Carter *et al.* 2012; Tyler *et al.* 2013), and we describe it briefly here. The first step in calculating CAPE coefficients is a reparametrization of the regression coefficients to obtain delta parameters ($\delta_{1}$ and $\delta_{2}$) which describe the degree to which the presence of one SNP influences the phenotypic effects of the other SNP:

$$\left[\begin{array}{c} \delta_{1}\\ \delta_{2} \end{array}\right]={\left[\begin{array}{cc} \beta_{1}^{1} & \beta_{2}^{1}\\ \beta_{1}^{2} & \beta_{2}^{2} \end{array}\right]}^{-1}\cdot \left[\begin{array}{c} \beta_{12}^{1}\\ \beta_{12}^{2} \end{array}\right]$$

These parameters are then translated into directional ($m_{12}$ and $m_{21}$) coefficients that self-consistently describe how the two SNPs influence one another:

$$\delta_{1}=m_{12}\left(1+\delta_{2}\right),\;\delta_{2}=m_{21}\left(1+\delta_{1}\right)$$

We estimated standard errors for these interaction terms by propagating standard error terms from least squares regression with second-order Taylor expansion on the regression parameters (Carter *et al.* 2012).

We estimated significance of the resulting model parameters through permutations with family-wise error rate estimation. We generated a null distribution as follows: ETs were randomly re-ordered relative to the genotypes, and we performed the single-SNP scan, and the pairwise scan as with the original data. We repeated this process until we had generated a null distribution with 1.5 million values. We corrected empirical *p* values using false discovery rate (FDR) (Benjamini and Hochberg 1995) and used a significance threshold of q<0.05.

To aid in visualizing the strength of each interaction we calculated a predicted additive effect of each pairwise SNP combination. These are shown in figure 2. We used the linear regression coefficients as shown in the following model.

$$y=\beta_{0}+{\text{x}}_{1}\beta_{1}+{\text{x}}_{2}\beta_{2}+{\text{x}}_{1}{\text{x}}_{2}\beta_{1,2}+\epsilon$$

We subtracted the intercept ($\beta_{0}$) from each term, and calculated the predicted additive effect as the sum of the two main effect *β* coefficients. Similarly, we defined the error as the sum of the standard errors of each group defined by the pairwise genotypes.

### Assignment of SNPs to genes

To assign each SNP in the final network to a gene, we downloaded SNP annotations from SNP Nexus (Dayem Ullah *et al.* 2018, 2013, 2012) and assigned each gene to the nearest or containing gene.

### Coexpression analysis

To explore the potential function of the interactions we identified, we used the Search-Based Exploration of Expression Compendium (SEEK) (Zhu *et al.* 2015). SEEK is a web tool (http://seek.princeton.edu) that searches across thousands of gene expression data sets to find genes that are co-expressed with a user-defined query gene or gene set. The search can be restricted to data sets in individual tissues or that have associated keywords, thus providing context for the co-regulation.

Based on the CAPE results, we analyzed two gene pairs: *WNT5A* and *RBMS3* in one query and *RBMS3* and *MSI2* in another. Using SEEK, we searched for co-regulated genes across all expression data sets. We also restricted our search to subsets of data sets to identify co-regulated genes in particular contexts (Table 2). For example, to look for tissue-specific co-regulation, we searched for genes co-regulated in all lung tissue data sets and also in all skin tissue data sets. Both tissues are relevant to SSc pathogenesis. However, in this study, we identified genetic interactions associated with lung function. We thus expected that genes co-regulated with the query genes in lung would better reflect the function of each interaction in this study.

In addition to tissue-specific searches, we also searched for genes co-regulated with our query genes in disease-specific data sets. To perform these searches, we restricted the search to data sets associated with key words, for example autoimmunity and fibrosis.

In each of these queries, we identified the genes whose expression was significantly correlated with the pair of query genes (p<0.05). These gene sets represent genes that were co-regulated with the query gene set across multiple tissues and disease contexts. We propose that functional enrichments of these gene sets are related to the function of the query gene pair in this disease cohort. To identify functional enrichment of each gene set, we used gProfileR (Reimand *et al.* 2016) (See workflow).

Among the significantly enriched terms, were many that are known to be related to SSc, for example terms relating to extracellular matrix organization and wound healing. We were interested in determining whether these sets of enriched GO terms enriched were related to SSc more than we would expect at random. To asses this, we downloaded a set of disease-GO term associations from the Comparative Toxicogenmoics Database (http://ctdbase.org/downloads/#phenotypediseases) (Davis *et al.* 2019, 2016). This database provided us with a set of GO terms that are associated with SSc.

From the database, we collected all GO terms associated with SSc, including Systemic Scleroderma, Diffuse Scleroderma, and Limited Scleroderma, and looked for overlap between these GO terms and the GO terms enriched in the SEEK analysis. To determine significance of the overlap between gene pair GO terms and SSc-associated GO terms, we performed Fisher’s Exact Test. Significance was assessed at p≤0.05/16.

For visualization purposes, we clustered SSc-related GO terms using REVIGO (Supek *et al.* 2011) (http://revigo.irb.hr).

Interestingly, although Wnt signaling has been shown to be involved in SSc, no Wnt signaling GO terms were associated with SSc by CTD. This indicates missing true associations between SSc and GO terms and suggests that the associations we calculated are conservative estimates.

### Data availability

Genotype and phenotype data are available from the Database of Genotypes and Phenotypes (DbGaP) (https://www.ncbi.nlm.nih.gov/gap) Accession: phs000357.v2.p1. Gene expression data are available from the Gene Expression Omnibus (GEO) (https://www.ncbi.nlm.nih.gov/geo/) Accession: GSE76808 A complete workflow including all code to generate results and figures is available here: https://github.com/annaLtyler/SSc_Epistasis_Workflow. Supplemental material available at figshare: https://doi.org/10.25387/g3.8300543.

## Results

### Trait descriptions

**Figure 1:**
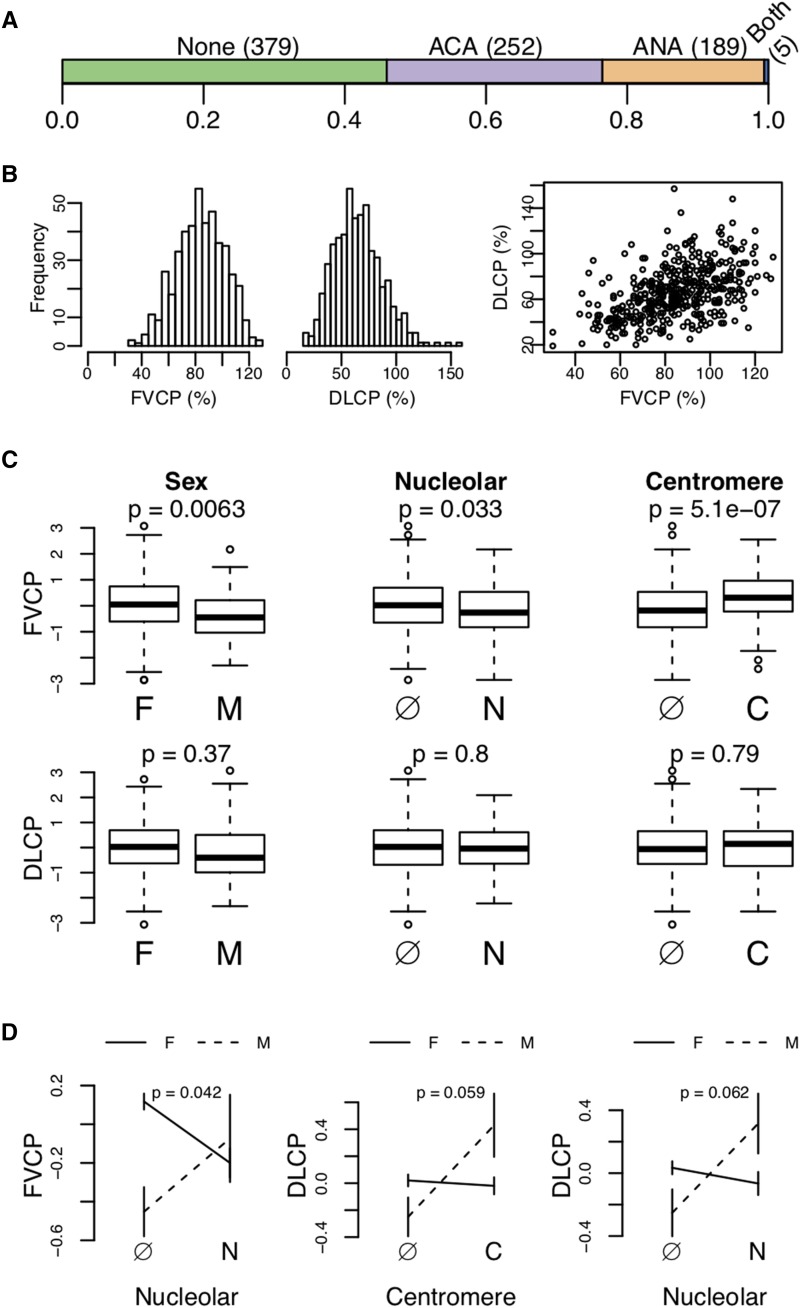
Trait distributions and correlations. (A) Colored bars show the proportion of patients that were negative for both autoantibodies (None), positive for either centromere (ACA) or nucleolar (ANA) autoantibodies, or positive for both (Both). (B) Histograms showing the distribution of FVCP (%) and DLCP (%). The distributions are roughly normal. The third panel shows the correlation between FVCP and DLCP. They were moderately correlated (r=0.48, $p=1.3\times 10^{-25}$). (C) Box plots show associations between lung traits and binary traits. Sex, nucleolar status, and centromere status all had significant effects on FVCP. None of the binary traits were significantly associated with DLCP. P values from two-tailed Student’s *t*-test (p<0.05) are reported. Autoantibody status is denoted as ANA (N), ACA (C) or absent (Ø). (D) These plots show interactions between binary traits that affect the lung traits. Sex and nucleolar status interacted significantly to influence FVCP (p≤0.05). Interactions between sex and autoantibody status on DLCP were not quite significant, but trended toward significance (p≈0.06).

We analyzed a cohort of 416 patients with lung function and autoantibody measurements (369 females and 47 males). The two lung traits, forced vital capacity (FVCP) and diffusion lung capacity (DLCP), were measured as a percentage of the total predicted by patient demographic parameters, such as age and weight (Agarwal *et al.* 2009). The autoantibodies were distributed as shown in Figure 1A. Roughly half the patients were negative for both antinucleolar autoantibodies (ANA) and anti-centromeric autoantibodies (ACA), and the other half of patients were positive for one or the other. Only five patients presented with both autoantibodies.

Both lung traits were roughly normally distributed, with maximum values greater than 100%, and the traits were modestly correlated (Pearson’s r=0.48, $p=1.3\times 10^{-25}$) (Figure 1B). We used rank Z normalization on both traits prior to performing association tests.

FVCP was influenced by sex and autoantibody status (Figure 1C). Males had significantly lower FVCP on average than females (two-taied Student’s *t*-test p=0.0063). The presence of the two autoantibodies had opposite effects. The presence of ANA significantly reduced FVCP (two-tailed Student’s *t*-test p=0.033), while the presence of ACA significantly improved FVCP (two-tailed Student’s *t*-test $p=5.1\times 10^{-7}$). Neither autoantibody nor sex had any significant association with DLCP (all two-tailed Student’s *t*-test p>0.05).

We also looked for interactions between the autoantibodies and sex that influenced the lung traits (Figure 1D). Presence of ANA and sex interacted nominally significantly to influence FVCP (p=0.042). In this interaction, females positive for ANA had lower FVCP on average than females negative for ANA. However, in males, the effect was the opposite. Males positive for ANA had higher FVCP on average than males negative for ANA. None of the other factor combinations had any significant effects on either FVCP or DLCP (all two-tailed Student’s *t*-test p>0.05); however sex and autoantibody status trended toward a significant interaction for DLCP. The presence of each autoantibody had very little effect on DLCP in females, but increased DLCP in males.

### Single-locus association identified four SNPs with significant main effects on ACA

The patients in this study were genotyped at 601,273 SNPs. However, because detection of genetic interactions can be confounded by low minor allele frequencies (MAF), we first filtered this data set to 243,662 SNPs with MAF ≥0.1. We then performed all single marker regressions (Materials and Methods). The purpose of this analysis was twofold. First it allowed us to determine whether the number of patients in this cohort was sufficient to recapitulate known associations between SNPs and SSc traits. Second, we used the *p* value distributions from this analysis to investigate whether population structure in this cohort might affect the SNP associations (Materials and Methods). There was no systematic effect of population structure in this cohort (Fig S1). We therefore did not use a correction for population structure in this analysis.

The SNP association tests identified significant main effects in the HLA region influencing the presence of ACA (Table 1). One of these SNPs (rs9275390) maps upstream of HLA-DBQ1 and was previously associated with positive ACA status in an SSc GWAS (Gorlova *et al.* 2011). Another SNP, rs660895, maps upstream of HLA-DRB1, and was previously associated with risk of the autoimmune disease Rheumatoid Arthritis (Plenge *et al.* 2007).

**Table 1:** Information about position of SNPs significantly associated with ACA (*p* < 0.01)

SNP	Chr	Position	Nearest Gene	Annot.	Dist. (bp)
rs4248166	6	32398644	BTNL2	intronic	
rs660895	6	32609603	HLA-DRB1	upstream	19755
rs7755224	6	32684540	HLA-DQB1	upstream	16157
rs9275390	6	32701379	HLA-DQB1	upstream	32996

### Multiple epistatic interactions influenced lung and autoantibody traits

**Figure 2:**
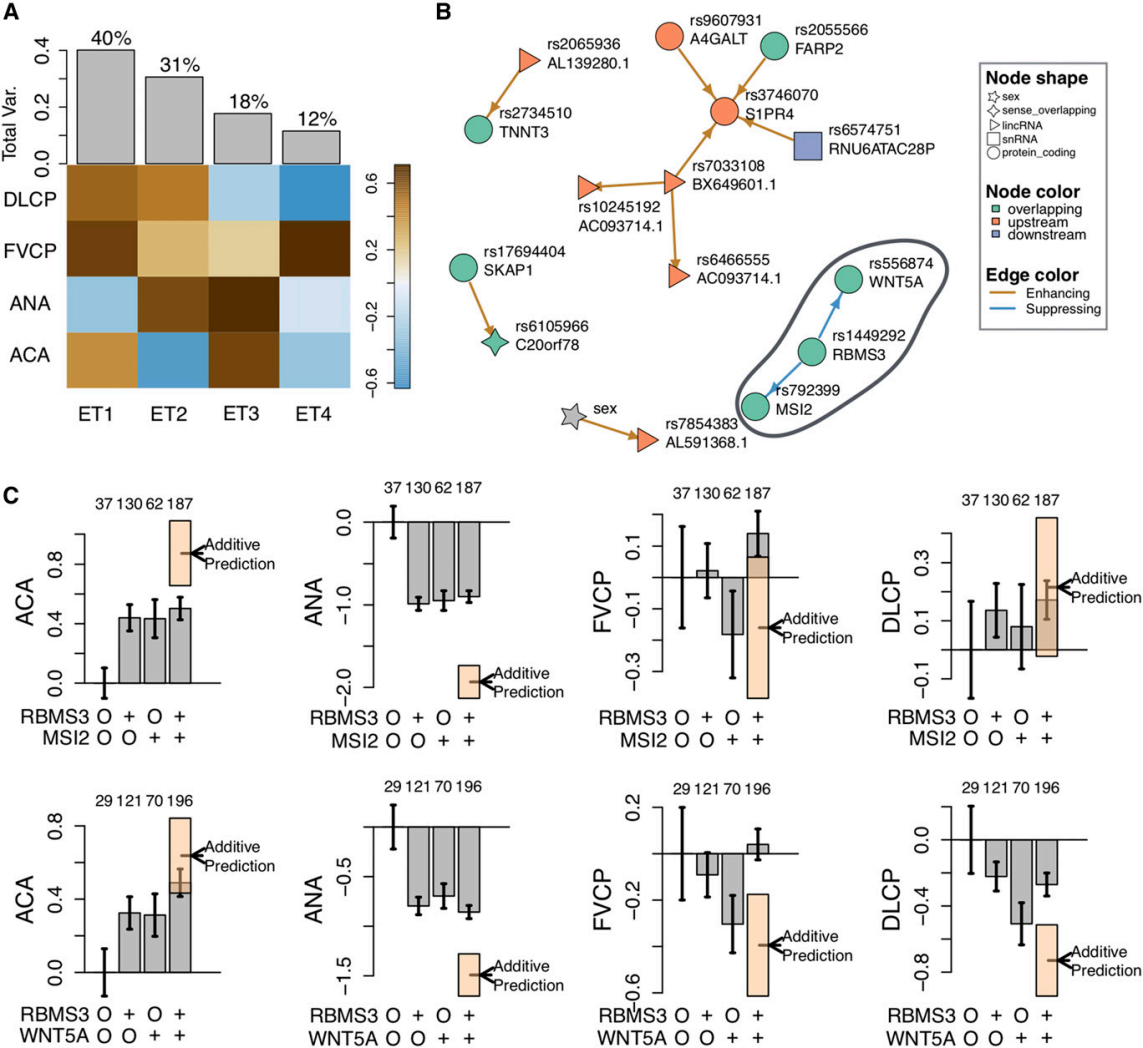
Genetic interactions influenced lung function and autoantibodies in SSc. (A) The relative contributions of each trait to each eigentrait (ET). Gray bars indicate how much overall variance each ET captures. We used the first two ETs, each of which contrasted one of the autoantibodies, with the other autoantibody and the lung function traits. These two ETs captured 71% of the overall trait variance. (B) The network of significant SNP-SNP interactions. Each node in the network represents one SNP. Each is labeled with the rs number, as well as with the name of the nearest gene. The color of each node indicates whether the SNP is upstream, downstream, or overlapping the gene. The shape of each node indicates the type of gene, protein coding, lncRNA, etc. The links between the nodes are colored to indicate whether each significant interaction was enhancing or suppressing. Three SNPs (rs556874, rs1449292, and rs792399) formed a three-node subnetwork we called the Wnt subnetwork (circled). Each of these SNPs overlaps a protein coding gene. (C) The effects of the Wnt subnetwork interactions on clinical traits. The *x*-axis in each plot indicates the four possible genotypes for each set of two SNPs. SNPs are labeled by their overlapping gene for clarity. The “O” indicates the reference genotype, and “+” indicates the alternate genotype. Gray bars indicate the mean trait value for the group of patients with the genotype indicated on the *x*-axis. The number above each bar indicates how many patients are in that group. Error bars show standard error. The additive prediction for each double alternate genotype is indicated by the dashed line and the error of the estimate is shown in the orange box. Gray bars that fall outside the orange box indicate interactions between the alleles. In A and C traits are abbreviated as follows: percent predicted forced vital capacity (FVCP) and percent predicted diffusion lung capacity (DLCP), antinucleolar autoantibodies (ANA), anticentromere autoantibodies (ACA).

We next performed pairwise SNP associations to identify interactions between SNPs. As CAPE is relatively computationally intensive, performing exhaustive pairwise testing ($>1.8\times 10^{11}$ tests) was not feasible. We thus filtered the SNPs to 1500 that were likely to participate in epistatic interactions influencing more than one trait (Materials and Methods). This number was selected such that the CAPE pipeline could be completed in about 24 hr. In addition, we decomposed the traits into orthogonal eigentraits (ETs) using singular value decomposition (SVD) (Figure 2A) (Materials and Methods). Traits that are moderately correlated may share common underlying biological processes. Using ETs concentrates signals from these underlying processes into single traits thereby increasing power to map them genetically. We selected the first two ETs for analysis. Each ET describes the contrast between one autoantibody and the two lung traits. After calculating CAPE coefficients associated with the two ETs, we rotated the coefficients back to trait space thus identifying interactions associated with all traits simultaneously. By selecting only two ETs we lost some biological information contained in the other two ETs, but we simultaneously gained power to identify interactions influencing the biological processes encoded by the first two eigentraits.

We identified a network of 11 significant genetic interactions involving 15 SNPs and the covariate sex (Figure 2B) (FDR≤0.05). The two most significant interactions (largest standardized effects) formed a single subnetwork among SNPs rs556874, rs1449292, and rs792399 (Figure 2B). This subnetwork was notable in that, not only did it contain the most significant interactions, but all three SNPs were intronic in protein coding genes *WNT5A*, *RBMS3*, and *MSI2*. The SNP rs556874 is located in the fourth intron of *WNT5A*, which is a member of the large family of Wnt ligands. Wnt signaling is a widely important family of signaling pathways integral to embryonic development, carcinogenesis, and many other processes. The SNP rs1449292 is located in an intron of *RBMS3*. This gene encodes a member of a small family of proteins that bind single-stranded RNA and DNA to regulate a wide range of biological processes. The SNP rs792399 is located in an intron of *MSI2* about which little is known. Although both *WNT5A* and *RBMS3* are both encoded on Chr 3, there is no LD between the interacting SNPs in this cohort ($R^{2}=0.004$). We focused on this subnetwork, which we refer to as the “Wnt subnetwork,” for the remainder of the analysis.

### Interactions in the Wnt subnetwork were less than additive

The interactions in the Wnt subnetwork were suppressing (Figure 2B). This means that in the presence of the alternate allele at one locus, the phenotypic effects of the interacting locus were suppressed. In this case, the suppressing interactions resulted less-than-additive interactions. These effects can be seen in Figure 2C. The *RBMS3-MSI2* interaction primarily affected the two autoantibody traits. The alternate alleles of the SNPs each had a negative effect on autoantibody presence. However, the effects of both alternate alleles together was redundant and did not decrease autoantibody presence beyond either of the individual alleles. The effects of this interaction on lung function were not different from additive.

The *RBMS3-WNT5A* interaction affected both lung function traits and ANA. The effects on ANA were similar to the effects of the *RBMS3-MSI2* interaction: individual alternate alleles decreased ANA incidence, but having two alternate alleles did not further decrease incidence beyond that seen for a single alternate allele. For both lung function traits, the *WNT5A* alternate allele had a large negative effect in the presence of the *RBMS3* reference allele, but this effect was completely suppressed in the presence of the *RBMS3* alternate allele.

### Expression of RBMS3 and WNT5A was dysregulated in SSc lung

**Figure 3:**
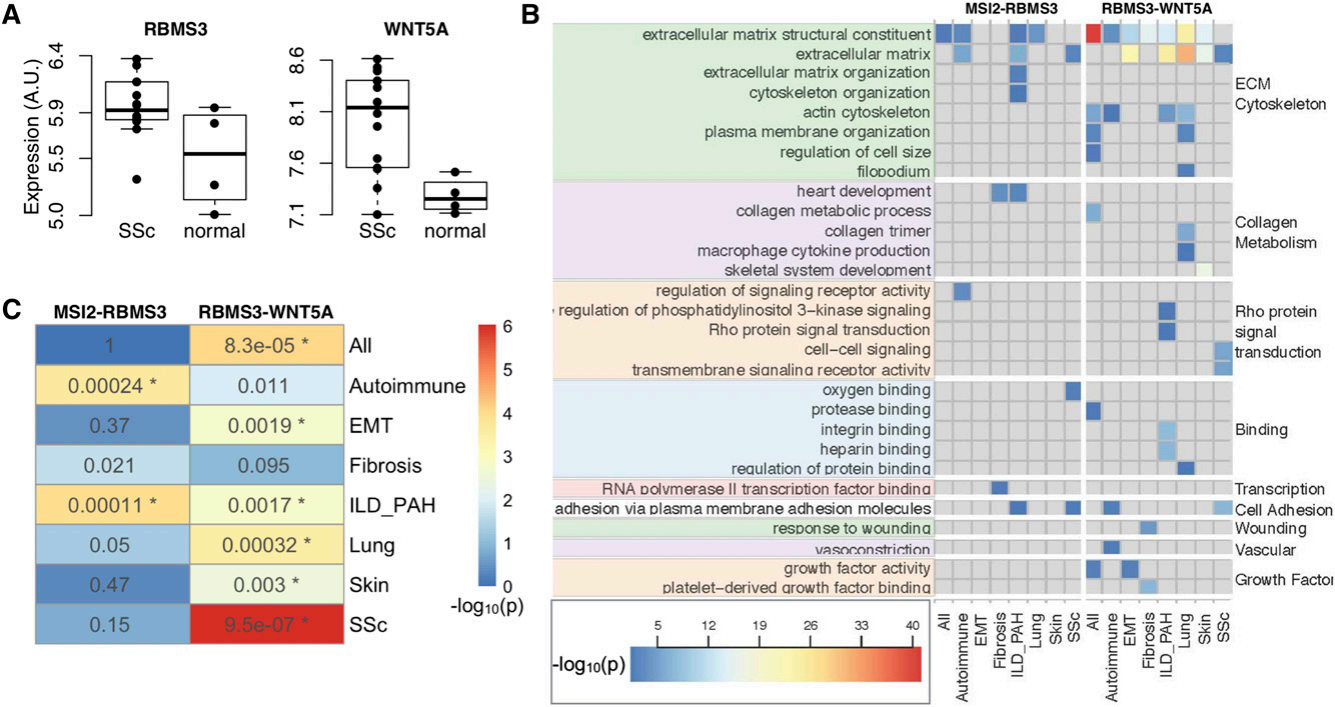
Expression of subnetwork genes in SSc. (A) Both *WNT5A* (two-tailed Student’s *t*-test p=0.011) and *RBMS3* (two-tailed Student’s *t*-test p=0.02) were significantly overexpressed in lung tissue from SSc patients with interstitial lung disease. (B) The $-log_{10}\left(p\right)$ for enriched GO terms in gene sets derived from SEEK (Materials and Methods). Each column shows results from a subset of the data sets on GEO. They are as follows: All - all data sets on GEO, Autoimmune - data sets studying autoimmunity, EMT - data sets studying epithelial to mesenchymal transition, Fibrosis - data sets studying fibrosis, ILD_PAH - data sets studying interstitial lung disease (ILD) or pulmonary arterial hypertension (PAH), Lung - data sets using lung tissue, Skin - data sets using skin tissue, SSc - data sets studying SSc. Each row shows the results for one GO term identified on the right-hand side. The GO terms are grouped functionally into nine groups identified by the GO term clustering algorithm REVIGO (Supek *et al.* 2011): ECM/cytoskeleton, collagen metabolism, Rho protein signaling transduction, Binding, Transcription, Cell Adhesion, Wound Repair, Vascular Function, and Growth Factors. The gene names at the top of the figure indicate that the left hand side of the figure shows results for the *RBMS3-MSI2* interaction, and the right side shows results for the *RBMS3-WNT5A* interaction. The legend indicates how the colors of the cells relate to the $-log_{10}\left(p\right)$ of the term enrichment. Only GO terms with fewer than 300 genes are shown. (C) Significance of overlap between gene pair-enriched GO terms and SSc-related GO terms. Fisher’s exact test *p* values are reported for each condition (rows) and each gene pair (columns). Asterisks indicate p≤0.5/16. Colors denote the $-log_{10}\left(p\right)$.

We investigated whether the candidate genes in the Wnt subnetwork were differentially expressed in SSc patient lung tissue (Materials and Methods) compared to control lung tissue. Differential expression between SSc lung tissue and control lung tissue suggests potential involvement in SSc pathogenesis. *WNT5A* (two-tailed Student’s *t*-test p=0.02) and *RBMS3* (two-tailed Student’s *t*-test p=0.011) had significantly higher expression in SSc lung tissue relative to healthy lung tissue (Figure 3A). Although we cannot assess the role of the genetic variants in expression level, differential expression supports the hypothesis that RBMS3 and WNT5A may be involved in SSc processes. We could not assess the differential expression of *MSI2*, as it was not present in the data set.

### Co-expressed genes were functionally enriched for processes related to SSc

To further investigate biological processes affected by the Wnt subnetwork, we searched across GEO for genes that were co-regulated with the gene pairs in the Wnt subnetwork. Genes with correlated expression often function together, and co-expression is frequently used to elucidate gene function (Eisen *et al.* 1998; Whitfield *et al.* 2002; Langfelder and Horvath 2008; Mahoney *et al.* 2015). We used this principle to identify possible functional roles of the interactions in the Wnt subnetwork. To investigate whether the interactions might be context-specific, we searched across multiple subsets of data sets in GEO (Materials and Methods). For example, to identify lung-specific functions of each gene pair, we searched for genes with correlated expression among data sets that were obtained from lung tissue. To identify functions that were related to autoimmunity, we searched only among data sets that analyzed autoimmune disease. All data set subsets are listed in (Table 2).

**Table 2:** The terms used to refine searches across gene expression data sets in GEO. Subsets were defined either by selecting a pre-defined subset (categorical) or by using key words (keyword) (Materials and Methods). The final column indicates how many data sets were included when subset criteria were applied

Subset	Method	Data sets
All	none	3402
Autoimmune	categorical	18
EMT	keyword	13
Fibrosis	categorical	39
ILD_PAH	keyword	4
Lung	categorical	239
Skin	categorical	136
SSc	keyword	6

In each of these data set subsets, we queried the *WNT5A-RBMS3* gene pair separately from the *RBMS3-MSI2* gene pair. For each query, we identified sets of genes whose expression was significantly correlated with the two query genes (p<0.05), and used the R package gProfileR (Reimand *et al.* 2016) to identify functionally enriched GO terms in each gene set. Multiple GO terms were significantly associated with the gene set from each query. For a complete list of the enriched terms, see Fig S2. To focus on terms that are strongly related to SSc, we used disease-GO term associations provided by the Comparative Toxicogenomic Database (Davis *et al.* 2019, 2016). These terms, and their enrichment *p* values across all queries are shown in Figure 3B.

Genes coexpressed with *RBMS3* and *WNT5A* were functionally enriched for multiple relevant processes, including ECM organization, wound repair, vasoconstriction, and growth factor activity.

Both gene pairs were significantly correlated with extracellular matrix (ECM) GO terms across multiple tissue types and conditions (Figure 3); however, only the *RBMS3-WNT5A* pair was significantly correlated with genes specifically involved in collagen metabolism indicating that this pair may affect ECM through effects on collagen, whereas the *RBMS3-MSI2* pair may influence ECM in a collagen-independent manner.

These enrichments for collagen metabolism appeared in lung tissue experiments, but not skin suggesting that the gene pair may be involved in collagen metabolism in a tissue-specific manner. Similarly, most significant enrichments associated with the *RBMS3-MSI2* interaction were concentrated in lung, lung disease, and autoimmune conditions, suggesting that this interaction may be specific to lung tissue, and may be aberrantly regulated in autoimmune conditions.

Fisher’s exact tests showed that many of the groups of GO terms enriched in genes co-expressed with the epistatic gene pairs were significantly related to SSc processes (Figure 3C). Genes co-expressed with the *RBMS3*-*WNT5A* pair were significantly SSc-related across more conditions than the genes co-expressed with *RBMS3* and *MSI2*. The patterns for the two gene pairs suggest that the *RBMS3-MSI2* interaction may be more involved in autoimmunity, whereas, the RBMS3-WNT5A interaction may be more involved specifically in lung disease and SSc-specific processes.

### Positive interaction also highlights promising candidates

Although we focus here primarily on the Wnt subnetwork, we also investigated whether any of the enhancing interactions in the genetic interaction network showed promise for follow-up. The most significant positive interaction in the network involved two additional variants located within or very near protein coding genes. This interaction was between rs2055566, which is located in an intron of FARP2, and rs3746070, which is located 736 bp upstream of S1PR4. These two variants had additive effects on all traits except DLCP. Neither variant had a significant main effect on DLCP, but individuals with both alternate alleles, had improved DLCP relative to other individuals (Fig S3).

## Discussion

We used a novel analytical approach to identify an epistatic interaction network influencing lung function in patients with SSc. In this approach we used three strategies to increase power to detect interactions in a patient cohort. First, we used a SNP filtering step to select the most highly epistatic and pleiotropic SNPs for further testing. This step reduced the number of statistical tests performed, but may also increase false positive rate due to the trait-based nature of the selection. We addressed this in part by using permutation-based p values and a stringent FDR cutoff (≤0.05). We also noted that none of the interactions identified by CAPE overlapped with the interactions identified by Matrix Epistasis, *i.e.*, all CAPE interactions were novel combinations of SNPs selected by the filtering step; however, the results reported are intended for discovery, and validation is required.

As a second strategy for increasing power to detect interactions, we combined information across multiple related traits: two autoantibody traits, and two lung function traits. And third, we decomposed the traits into orthogonal eigentraits and used only the top two eigentraits in our study. This step reduced noise and captured mappable signals that related to all four traits. Thus, using Matrix Epistasis to pre-filter SNPs combined with CAPE allowed us to identify genetic interactions in a relatively small human cohort. Because of the increased false positive rate imposed by the SNP filtering step, the results reported here are primarily for discovery and hypothesis generation. Although the genes identified are supported by the literature as discussed below, the interactions between them need to be replicated in an independent cohort.

The resulting genetic interaction network contained a notable subnetwork consisting of two interactions between three SNPs. Because the interactions in this network were highly significant and because all three SNPs were located within protein-coding gene bodies, we focused on this subnetwork for further analysis. We also briefly investigated the most significant positive interaction between SNPs that were also associated protein coding genes, FARP2 and S1PR4. Each of the candidates implicated by these interactions has been associated either directly with SSc or with SSc-related processes, which speaks to the reliability of this analysis.

The two genes participating in the positive interaction have been more directly linked to SSc than the genes in the Wnt subnetwork. S1PR4, for example, encodes the sphingosine-1-phosphate receptor 4, and sphingosine-1-phosphate well is known to affect SSc pathogenesis. This signaling phospholipid is elevated in the serum of SSc individuals and is capable of producing many of the abnormalities seen in SSc (Pattanaik and Postlethwaite 2010; Tokumura *et al.* 2009). Sphingosine-1-Phosphate Receptor 5 was found to modulate early fibrogenesis in a mouse model of SSc (Schmidt *et al.* 2017), and the selective S1P1 receptor modulator cenerimod has been shown to attenuate lung and skin fibrosis in two different mouse models of SSc (Kano *et al.* 2019). FARP2 encodes FERM, RhoGEF and pleckstrin domain protein 2 and is involved in semaphorin signaling. Dysregulation of semaphorin signaling has been associated with antibody production, disease type, thickening of skin, disease duration, and active inflammation in SSc patients (Besliu *et al.* 2011; Rimar *et al.* 2015).

The genes in the three-gene Wnt subnetwork also have support in the literature for SSc involvement. We focus the discussion primarily on these genes to highlight the possible connections between genetic interactions and functional relatedness. Although none of the genes in the Wnt subnetwork had previously been associated with lung function in SSc, they have all been implicated in lung disease, as well as in molecular processes that are known to be involved in SSc pathogenesis.

*WNT5A*, and Wnt signaling more generally, have been implicated in many processes known to be involved in SSc pathogenesis (Tsou and Sawalha 2017; Wei *et al.* 2011; Katsumoto *et al.* 2011; Lafyatis 2012), such as angiogenesis (Huang *et al.* 2005), keratinocyte differentiation and inflammation signaling (Wang *et al.* 2018), and fibroblast proliferation (Vuga *et al.* 2009). *WNT5A* has also been linked to cellular transdifferentiation, a family of processes by which differentiated cell types dedifferentiate and redifferentiate into another cell type. Transdifferentiation processes are critical for embryonic development, wound healing and tissue regeneration (Kalluri and Weinberg 2009). However, when they are dysregulated, these processes contribute to metastases in cancer, and widespread fibrosis seen in fibrotic diseases (Kalluri and Weinberg 2009). There are three types of transdifferentiation that have been linked to fibrosis in SSc: epithelial to mesenchymal transition (EMT) (Postlethwaite *et al.* 2004), endothelial to mesenchymal transition (EndoMT) (Manetti *et al.* 2017) and adipocyte-myofibroblast transition (AMT) (Marangoni *et al.* 2015).

Each of these processes has been hypothesized to be a source of excess myofibroblasts in SSc tissues. Myofibroblasts differentiate from fibroblasts in response to signaling from TGF-*β*, as well as other growth factors and cytokines. In healthy individuals, myofibroblasts play an important role in wound healing. They contract to close wounds and deposit ECM to seal wounds and to provide a scaffold for the re-establishment of epithelial tissue. In SSc and other fibrotic disease however, there are increased numbers of myofibroblasts with a concomitant increase in the deposition of extracellular matrix, which causes tissue stiffening (Postlethwaite *et al.* 2004; Nakamura and Tokura 2011). EMT has been directly implicated in lung fibrosis in mice (Kim *et al.* 2006; Hashimoto *et al.* 2004), and may be an important source of myofibroblasts in SSc skin (Nakamura and Tokura 2011). Wnt signaling, and *WNT5A* in particular, has been shown to mediate EMT in melanoma (Dissanayake *et al.* 2007) and in lung cancer (Wang *et al.* 2017).

Endothelial to mesenchymal transition (EndoMT) has also been proposed to be a source of myofibroblasts in SSc (Piera-Velazquez *et al.* 2011; Manetti *et al.* 2017; Jimenez and Piera-Velazquez 2016). Originally thought only to occur in developing embryos, EndoMT has been demonstrated to occur in adult cattle (Arciniegas *et al.* 1992) and mice (Zeisberg *et al.* 2007). TGF-*β*-induced EndoMT has since been shown to be involved in the bleomycin model of pulmonary fibrosis as well as SSc-associated pulmonary hypertension (Jimenez 2013; Jimenez and Piera-Velazquez 2016). Furthermore, there is evidence to suggest that Wnt signaling may mediate EndoMT (Jimenez and Piera-Velazquez 2016).

The third transdifferentiation process to be proposed as a source of myofibroblasts in SSc is AMT (Marangoni *et al.* 2015). Marangoni *et al.* (2015) showed that when adipose-derived stem cells from healthy donors were incubated with TGF-*β*, they lost markers of adipocytes, and gained markers of myofibroblasts. In this experiment *WNT5A* was strongly upregulated in the transitioning cells.

Like *WNT5A*, *RBMS3* has been linked to transdifferentiation. Upregulation of *RBMS3* inhibits EMT in lung cancer through the inhibition of canonical Wnt signaling (Yang *et al.* 2018; Liang *et al.* 2015). By downregulating MMP2 and *β*-catenin, *RBMS3* also inhibits microvessel formation (Chen *et al.* 2012). Loss of microvessels and reduced angiogenesis are hallmarks of SSc (Manetti *et al.* 2010; Distler *et al.* 2004). *RBMS3* may also directly influence collagen synthesis (Penkov *et al.* 2000; Fritz and Stefanovic 2007), which is highly upregulated in SSc. Although *RBMS3* has not been directly linked to SSc, SNPs in *RBMS3* have been associated with risk of another autoimmune disease affecting connective tissue called Sjögren’s syndrome (Song *et al.* 2016).

Of the three genes in the Wnt subnetwork, the least is known about *MSI2*, and it is most studied in the context of cancer and its influence on EMT (He *et al.* 2014; Kudinov *et al.* 2016). *MSI2* is upregulated in metastatic-competent lung cancer cell lines, and aggressively metastatic patient tumors have upregulated *MSI2* (Kudinov *et al.* 2016). Conversely, depletion of *MSI2* resulted in reduced metastatic potential (Kudinov *et al.* 2016). As mentioned previously, EMT may be an important process in SSc pathogenesis. *MSI2* is also related to Wnt signaling and may influence hepatocellular carcinoma outcomes through dysregulation of Wnt signaling (Wang *et al.* 2015).

Thus, all three genes in the Wnt subnetwork have been implicated in mediating the transdifferentiation process of epithelial to mesenchymal transition (EMT). Both EMT (Willis and Borok 2007) and EndoMT (Jimenez and Piera-Velazquez 2016) are known to take place in the lung and have demonstrated roles in SSc (Jimenez 2013; Postlethwaite *et al.* 2004). These results suggest that EMT, or another similar transdifferentiation, may play a role in lung disease pathogenesis and lung function in SSc patients, and that genes in the Wnt subnetwork we identified here may be involved.

Furthermore, all three genes in the Wnt subnetwork have been shown to be associated with Wnt signaling. These pathways are known to be important both in lung development, and in lung fibrosis in SSc (Pongracz and Stockley 2006; Lam *et al.* 2011). Wnt signaling, both canonical (*β*-catenin mediated) and non-canonical (not *β*-catenin mediated), has been shown to drive EMT. Upregulation of *WNT5A* increases EMT in lung cancer (Wang *et al.* 2017) possibly through stimulation of the non-canonical PKC Wnt pathway (Dissanayake *et al.* 2007). Similarly, upregulation of *MSI2* is an indicator of increased metastatic potential in lung cancer (Kudinov *et al.* 2016) and hepatocellular carcinoma (Kudinov *et al.* 2016), and is proposed to stimulate EMT through stimulation of canonical Wnt signaling (Wang *et al.* 2015). *RBMS3* has been shown to reduce EMT and metastatic capacity of lung cancer cells (Liang *et al.* 2015) and reduces EMT in gastric cancer cells through suppression of canonical Wnt signaling (Yang *et al.* 2018). Taken together, these results suggest that the Wnt subnetwork we identified here may mediate cellular transdifferentiation in the SSc lung through regulation of Wnt signaling.

Importantly, the Wnt subnetwork includes not only three novel candidate genes, but also the genetic interactions between them. These interactions suggest functional relatedness, which is broadly supported through connections in the literature with EMT and Wnt signaling. But we can also make more direct connections between the specific genes by using a more agnostic gene expression-based approach. We looked at genes that were co-expressed with each pair of subnetwork genes across multiple different tissue and disease contexts, including lung tissue, lung disease, and skin tissue. We found that the gene pairs were associated with multiple processes related to SSc pathogenesis, such as ECM organization, growth factor activity, and vascular abnormalities. By comparing the enrichments related to the two interactions, we can generate hypotheses about their specific involvement. For example, both gene pairs were co-regulated with genes involved in ECM-related GO processes; however only genes co-expresed with *RBMS3* and *WNT5A* were associated with collagen metabolism, suggesting that *RBMS3* and *MSI2* together may influence a distinct aspect of ECM.

Interestingly, functional enrichment of co-expressed genes also appeared to be context-specific. SSc-related functional enrichments were seen predominantly in the lung disease (ILD_PAH) data sets, as well as in autoimmunity data sets, as opposed to skin. We interpret this to mean that both genetic interactions may be tissue- and disease-specific. It is possible that these interactions were only detectable because we looked specifically at lung function outcomes in an SSc cohort. Thus, the interactions we identified may not be related to skin pathology. Validation experiments, therefore, should focus on lung tissue.

We further hypothesize that these interactions may be particularly important to lung function in a disease state. All three SNPs we identified have high minor allele frequencies in the general population (rs556874 MAF ≈0.4, rs1449292 MAF ≈0.5, and rs792399 MAF ≈0.4) (Sherry *et al.* 2001). We hypothesize that these SNPs are unlikely to have any measurable effect on lung function in healthy individuals, but that the interactions between them become important in disease. We further hypothesize that because these alleles are so common, they may not increase the risk of developing SSc, but that they affect the course of the disease once established. We performed our genetic analysis on a cohort of patients with SSc that did not include controls. Although a cohort containing only affected individuals may have few individuals, thereby reducing power to detect interactions, we assert that this type of case-only study is important in detecting disease-specific interactions that would not be detectable in a data set that included controls.

These interactions may be important considerations in personalized medicine. Whereas genetic main effects are averaged across all genetic backgrounds, genetic interactions may identify clinically relevant subgroups of patients based on allele combinations. For example, in this study we found that the minor allele of rs556874 in WNT5A had substantially reduced lung function, but only in patients with the major allele of rs1449292 in RBMS3. With this knowledge, this subset of patients can be monitored more closely for lung disease and perhaps provided with treatments for lung disease earlier in the course of their disease.

In this study we have taken a systems approach to identifying potential SSc-related processes by analyzing genetic interactions that influence multiple disease traits simultaneously. By addressing the complexity of this disease in our models we increased power to detect novel interactions among highly promising candidate genes. The interactions, furthermore, may be disease- and tissue-specific. Identifying such specific interactions may provide a promising avenue forward in drug target discovery. The genes identified in this study are all widely expressed, and targeting them broadly may result in unintended effects. However, because evidence suggests that the interactions between the genes may be tissue- and disease-specific, perhaps the interactions themselves are better targets than the genes. For example, rather than attempting to reduce *WNT5A* expression globally, a specific targeting of its interaction with *RBMS3* may provide a novel approach to treating SSc-related lung disease without widespread off-target effects. Known genetic interactions may also help predict disease course in individual SSc patients. If it is known, for example, that a patient has the reference *RBMS3* allele and the alternate *WNT5A* allele, this might indicate a highly likelihood of developing severe SSc-related lung disease, and testing and treatment of lung disease could be started early and aggressively. Genetic interactions offer a rich source of information for the understanding and treatment of disease, and new computational methods that take genetic and physiological complexity into account have the potential to build on the past success of GWAS and to piece together connections between individual findings that are currently isolated from one another.
